# A genome-wide association study of atopic dermatitis identifies loci with overlapping effects on asthma and psoriasis

**DOI:** 10.1093/hmg/ddt317

**Published:** 2013-07-25

**Authors:** Stephan Weidinger, Saffron A.G. Willis-Owen, Yoichiro Kamatani, Hansjörg Baurecht, Nilesh Morar, Liming Liang, Pauline Edser, Teresa Street, Elke Rodriguez, Grainne M. O'Regan, Paula Beattie, Regina Fölster-Holst, Andre Franke, Natalija Novak, Caoimhe M. Fahy, Mårten C.G. Winge, Michael Kabesch, Thomas Illig, Simon Heath, Cilla Söderhäll, Erik Melén, Göran Pershagen, Juha Kere, Maria Bradley, Agne Lieden, Magnus Nordenskjold, John I. Harper, W.H. Irwin Mclean, Sara J. Brown, William O.C. Cookson, G. Mark Lathrop, Alan D. Irvine, Miriam F. Moffatt

**Affiliations:** 1 Department of Dermatology, Venereology and Allergy, University Hospital Schleswig-Holstein and; 2 Christian-Albrechts-University of Kiel, Kiel, Germany; 3 National Heart and Lung Institute, Imperial College, LondonSW3 6LY, UK; 4 Fondation Jean Dausset—Centre d’Étude du Polymorphisme Humain, Paris, France; 5 Department of Dermatology, Chelsea and Westminster Hospital, Fulham Road, London SW10 9NH, UK; 6 Department of Epidemiology and; 7 Department of Biostatistics, Harvard School of Public Health, Boston, MA, USA; 8 Department of Statistics, University of Oxford, Oxford OX1 3TG, UK; 9 National Children's Research Centre and; 10 Department of Paediatric Dermatology, Our Lady's Children's Hospital, Dublin 12, Ireland; 11 Royal Hospital for Sick Children, Yorkhill, Glasgow G3 8SJ, UK; 12 Department of Dermatology, University Hospital Schleswig-Holstein, Campus Kiel, Kiel, Germany; 13 Department of Dermatology and Allergy, University of Bonn, Bonn, Germany; 14 Department of Clinical Medicine, Trinity College Dublin, Dublin, Ireland; 15 Dermatology Unit, Department of Medicine Solna, Karolinska Institutet and Karolinska University Hospital, Stockholm, Sweden; 16 Department of Molecular Medicine and Surgery, Center for Molecular Medicine and; 17 Department of Biosciences and Nutrition and; 18 Institute of Environmental Medicine and; 19 Department of Biosciences and Nutrition, Karolinska Institutet, Stockholm, Sweden; 20 Department of Pediatric Pneumology and Allergy, University Children's Hospital Regensburg (KUNO), Campus St. Hedwig, Germany; 21 Institute of Epidemiology, Helmholtz Zentrum Munich, Munich, Germany; 22 Hannover Unified Biobank, Hannover Medical School, Hannover, Germany; 23 CEA/Centre National de Genotypage, 91057 Evry, France; 24 Sach's Children's Hospital, Stockholm, Sweden; 25 Science for Life Laboratory, Stockholm, Sweden; 26 Research Programs Unit, University of Helsinki and Folkhälsan Institute of Genetics, Helsinki, Finland; 27 Department of Immunobiology and Dermatology, UCL Institute of Child Health, London, UK; 28 Department of Paediatric Dermatology, Great Ormond Street Hospital for Children, London, UK; 29 Dermatology and Genetic Medicine, College of Life Sciences, and College of Medicine, Dentistry and Nursing, University of Dundee, DundeeDD1 5EH, UK; 30 McGill University and Genome Quebec Innovation Centre, McGill University, Montreal, Canada

## Abstract

Atopic dermatitis (AD) is the most common dermatological disease of childhood. Many children with AD have asthma and AD shares regions of genetic linkage with psoriasis, another chronic inflammatory skin disease. We present here a genome-wide association study (GWAS) of childhood-onset AD in 1563 European cases with known asthma status and 4054 European controls. Using Illumina genotyping followed by imputation, we generated 268 034 consensus genotypes and in excess of 2 million single nucleotide polymorphisms (SNPs) for analysis. Association signals were assessed for replication in a second panel of 2286 European cases and 3160 European controls. Four loci achieved genome-wide significance for AD and replicated consistently across all cohorts. These included the epidermal differentiation complex (*EDC*) on chromosome 1, the genomic region proximal to *LRRC32* on chromosome 11, the *RAD50/IL13* locus on chromosome 5 and the major histocompatibility complex (MHC) on chromosome 6; reflecting action of classical HLA alleles. We observed variation in the contribution towards co-morbid asthma for these regions of association. We further explored the genetic relationship between AD, asthma and psoriasis by examining previously identified susceptibility SNPs for these diseases. We found considerable overlap between AD and psoriasis together with variable coincidence between allergic rhinitis (AR) and asthma. Our results indicate that the pathogenesis of AD incorporates immune and epidermal barrier defects with combinations of specific and overlapping effects at individual loci.

## INTRODUCTION

Atopic dermatitis (AD) is a chronic relapsing inflammatory skin disease that may affect 20% of children and 3% of adults. Up to 60% of children with severe AD will develop asthma (1), and infantile AD is a strong predictor of subsequent asthma development (2). These observations are consistent with the genetic correlation of 0.35–0.55 (3,4) found between these two strongly heritable (5–10) diseases, and indicates some shared elements of aetiology.

Despite this apparent overlap, few genes implicated in AD have been confirmed to play a role in asthma. The positionally cloned (11,12) and highly replicated (13) *FLG* locus may contribute towards the pathogenesis of asthma, but only within the context of AD (11,14). *FLG* mutations have not been found to be a risk factor for asthma alone, suggesting that ‘AD plus asthma’ is a genetically distinct form of asthma or a specific endophenotype of AD (11,14,15). In contrast, far greater levels of genomic coincidence have been observed between AD and psoriasis (1), another common familial inflammatory skin disease. While not mutually exclusive, AD and psoriasis rarely co-exist. They share a number of similar features, particularly in the chronic phase of AD, including epidermal hyperplasia and altered terminal differentiation of keratinocytes (16,17), as well as a number of highly divergent features, such as the level of cornification and susceptibility towards recurrent bacterial, viral or fungal infections.

Genome-wide association studies (GWAS) have confirmed the association between AD and the *FLG* locus (18–21) and specified additional contributory loci at 2q12 (21), 3p21.33 (21), 3q13.2 (21), 5q22.1 (19,21), 5q31.1 (18,21), 6p21.3 (21), 7p22 (21), 10q21.2 (21), 11p15.4 (21), 11q13.1 (18,21), 11q13.5 (18,20,21), 19p13.2 (18,21) and 20q13 (18,19,21). To date, more than half of these loci have only been observed in Japanese populations. As yet, none of these GWAS have specifically examined the differing genetic contribution of individual loci to the AD endophenotypes ‘AD plus asthma’ and ‘AD no asthma’ on a genome-wide scale.

**Figure 1.**
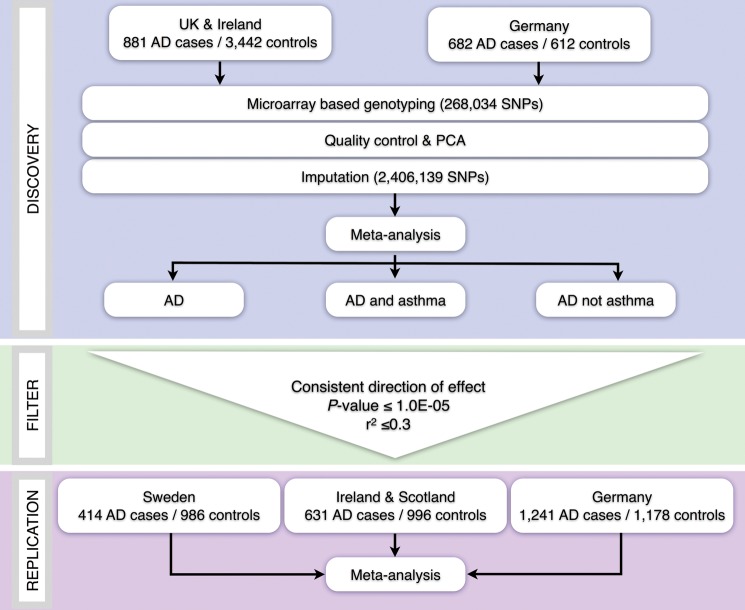
Schemata of study design.

In this study, we consequently have set out to map the genetic architecture of AD in a Northern European population and to explore the genetic overlap between AD and asthma in the same dataset (Fig. 1). Using existing evidence from GWAS of psoriasis, we also sought to identify genetic communalities between these two common inflammatory skin diseases.

## RESULTS

The discovery phase comprised 1563 childhood onset moderate-severe AD cases of European descent recruited through secondary and tertiary referral centres and 4054 population controls of matched ancestry. The subject set incorporated independent cohorts from the UK and Ireland (881 cases, 3442 controls) and Germany (682 cases, 612 controls). Overall, 36.4% of the cases also had a doctor diagnosis of asthma (Table 1; Supplementary Material, Table S1). A total of 268 034 observed and 2 406 139 imputed single nucleotide polymorphisms (SNPs) passed quality control filters. There was little evidence of inflation of the test statistic due to population stratification, but a marked enrichment for significant values at the tail of the distribution. Genomic control (GC) inflation factors for the UK and German portions of the cohort with 10 principal components as covariates were 0.995 and 1.010, respectively, for AD, 1.007 and 1.005 for AD and asthma, and 0.977 and 0.997 for AD not asthma. For the data with GC factor ≤1.00, no further adjustments were made. For the data with GC factor >1.00, genomic control was applied prior to meta-analysis.

**Table 1.** Sample and population summary

Variable	European ancestry discovery sets			European ancestry replication sets			
	UK and Irish	German	Total	Irish and Scottish	German	Swedish	Total
*n* (cases/controls)	881/3442	682/612	1563/4054	631/996	1241/1178	414/986	2286/3160
Mean age (cases/controls)	7.2 (8.8)/ 39.2 (13.5)	25.2 (14.1)/9.6 (0.7)	15.0 (14.5)/34.7 (16.4)	2.8 (3.6)/38.3 (12.7)	21.4 (16.6)/26.7 (17.3)	29.2 (13.9)/8.3 (0.49)	19.0 (16.8)/24.5 (17.6)
% Male cases	57.7	39.1	49.6	59.5	45.7	40.3	47.8
% Male controls	51.2	49.3	51.0	57.8	43.9	50.9	50.4
% Asthma^a^	37.0	35.6	36.4	15.0	31.8	45.4	29.9
Genotyping	Illumina 300 K/610K	Illumina 300K	–	TaqMan	Sequenom	Sequenom and TaqMan	–

^a^Based on the individuals with data available.

**Figure 2.**
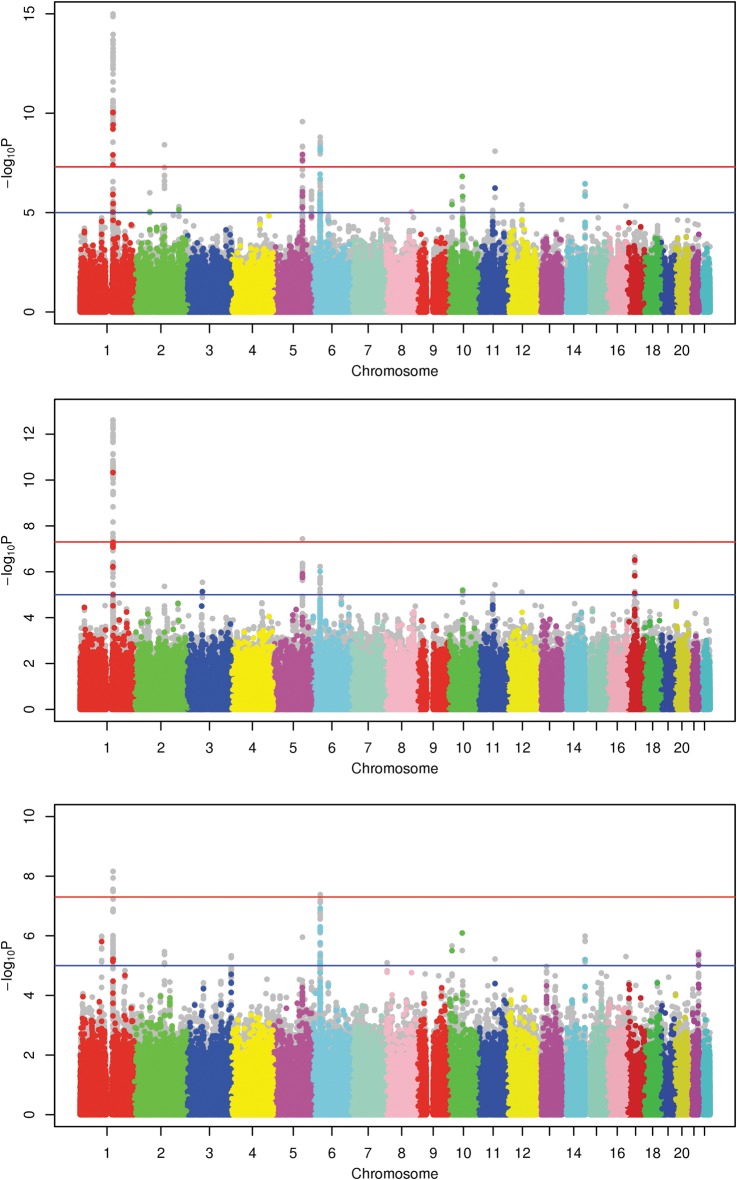
Manhattan plot of GWAS results. Manhattan plots relating to each strata of the GWA analysis presented in the following order: AD (all), AD and asthma, AD no asthma. The threshold for genome-wide significance *(P* ≤ 5.0E−08) is shown on each plot as a solid red line. A suggestive threshold of 1.0E−05 is shown as a solid blue line. Abbreviations: −log10(*P-*value), the base 10 logarithm of the probability (*P*) values; AD, atopic dermatitis.

**Figure 3.**
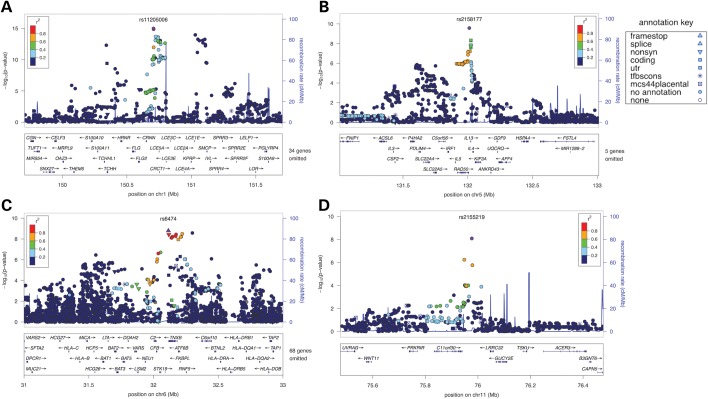
Detailed maps of regions showing robust evidence of association, including sites which replicate successfully across all cohorts, and the established 1q21 locus. Points are coloured according to the extent of LD (as measured by their *r*^2^ values) as indicated by the colour scale. The presence of association peaks with a dark blue colour indicates independent association. The shape of individual points reflects the UniSNP database annotation (see legend and http://research.nhgri.nih.gov/tools/unisnp/?Rm=ohelp). Mb, megabase; Chr, chromosome; nonsyn, nonsynonamous; utr, untranslated region; tfbscon, conserved, predicted transcription factor binding side; mcs44placental, conserved, placental mammals. (**A**) Chromosome 1q21. (**B**) Chromosome 5q31. (**C**) Chromosome 6p21. (**D**) Chromosome 11q13.

Contiguous regions of association to AD (irrespective of asthma status) were observed on chromosomes 1q21 (top SNP *rs11205006*, *P* = 1.02E−15), 2q22 (top SNP *rs1469621*, *P* = 3.88E−09), 5q31 (top SNP *rs2158177*, *P* = 2.65E−10), 6p21 (top SNP *rs6474*, *P* = 1.61E−09) and 11q13 (top SNP *rs2155219*, *P* = 8.17E−09) (Fig. 2). The linkage disequilibrium (LD) patterns across the regions were consistent with single effect loci on chromosomes 2q22 and 11q13, and multi-effect loci on chromosomes 1q21, 5q31 and 6p21 (Fig. 3). Independence among association signals at each of these sites was tested explicitly via three cycles of conditional logistic regression, sequentially incorporating the strongest associations as covariates. In order to increase the minimum observed *P*-value >0.0001, it was necessary to condition on three markers on chromosome 1q21 (*rs11205006*, *rs12086263* and *rs17670505*), two markers on chromosome 5q31 (*rs2158177* and *rs157573*) and 6p21 (*rs6474* and *rs3134929*), and one marker on chromosome 2q22 (*rs1469621*) and 11q13 (*rs2155219*).

The association signal on chromosome 1 coincided with the *FLG* locus (13), the most robust known genetic risk factor for AD (22), and one of the strongest known genotype–phenotype associations of any complex trait. Independence among the chromosome 1 SNPs and the most common *FLG* AD risk alleles (R501X and 2282del4) was assessed by logistic regression (Supplementary Material, Table S2). Known *FLG* AD risk alleles accounted for the majority of variance observed at this site with no significant residual effects attributable to other associated SNPs. The association at this site was more pronounced in individuals with both AD and asthma, as compared with those with AD alone (Fig. 2). This observation is consistent with data, suggesting that the risk for asthma conferred by *FLG* variants is limited to asthma occurring within the context of AD (23,24).

Inclusion of asthma disease status into the GWAS model (AD plus asthma, AD no asthma) did not lead to the identification of any additional loci significant at a genome-wide threshold (*P* ≤ 5.0E−08, Fig. 2). All 5 loci achieved peak significance when asthma status was not taken into account in the model most likely reflecting increased power through increased case numbers. Nevertheless, considerable variability was observed between loci; with the loci on chromosomes 1 and 5 achieving markedly greater significance in the ‘AD plus asthma’ group compared with the ‘AD no asthma’ group. The reverse scenario was observed for chromosome 6 (Fig. 2).

We sought replication of our findings by selecting the highest ranking SNPs with a consistent direction of effect in UK–Irish and German cohorts from the GWA discovery stage (*P* < 1.0E−05). Multiple SNPs ranked high from a single chromosomal region were filtered to exclude those in LD (*r*^2^ > 0.3) with the highest ranking SNP. Potentially functional SNPs from LD bins were retained. SNPs from the known *FLG* locus on chromosome 1 were not included due to prior replication by others. A total of 47 SNPs (Supplementary Material, Table S3) were carried forward to the replication cohorts, which contained 2286 cases and 3160 controls derived from Ireland, Scotland, Sweden and Germany.

Sixteen SNPs from three different genomic regions showed evidence of replication after Bonferroni correction for the 47 tests (*P* < 0.001). Associations were with the same allele and in the same direction as the discovery phase. Fourteen of the 16 SNPs achieved genome-wide significance in a meta-analysis incorporating all study collections (*P* < 5.0E−08, Table 2). These SNPs were positioned on chromosomes 5q31 (5 SNPs), 6p21 (7 SNPs) and 11q13 (2 SNPs).

**Table 2.** Association results of the GWAS screen and replication studies for AD

*SNP*	Chr	Pos	A1	A2	Gene	Screen^a^		Replication^b^		Combined^c^	
						OR	P	OR	P	OR	P
*rs2897443*	5	131957493	T	G	*RAD50*	1.350 (1.196–1.525)	1.27E−06	1.284 (1.171–1.408)	1.20E−07	1.308 (1.215–1.407)	8.95E−13
*rs6871536*	5	131997773	T	C	*RAD50*	0.738 (0.655–0.832)	7.27E−07	0.747 (0.678–0.822)	3.42E−09	0.743 (0.69–0.799)	2.11E−15
*rs2158177*	5	132011957	A	G	*RAD50/IL13*	0.611 (0.524–0.712)	2.65E−10	0.738 (0.672–0.811)	2.49E−10	0.704 (0.634–0.782)	5.90E−11
*rs1295686*	5	132023742	T	C	*IL13*	1.420 (1.255–1.606)	2.66E−08	1.362 (1.24–1.496)	9.80E−11	1.383 (1.283–1.49)	1.65E−17
*rs20541*	5	132023863	A	G	*IL13*	1.426 (1.260–1.613)	1.79E−08	1.365 (1.202–1.55)	1.56E−06	1.39 (1.278–1.512)	1.93E−14
*rs2251396*	6	31472686	A	G	*HLA-B*	1.325 (1.178–1.490)	2.65E−06	1.23 (1.109–1.365)	1.09E−04	1.267 (1.174–1.368)	1.28E−09
*rs2844509*	6	31618903	A	G	*BAT1*	1.345 (1.184–1.529)	5.64E−06	1.624 (1.249–2.112)	3.00E−04	1.312 (1.215–1.417)	4.33E−12
*rs9368699*	6	31910520	T	C	*C6orf48*	1.894 (1.432–2.505)	7.61E−06	1.531 (1.201–1.952)	5.81E−04	1.677 (1.396–2.014)	3.19E−08
*rs12198173*	6	32134786	A	G	*TNXB*	0.649 (0.539–0.782)	5.56E−06	0.676 (0.571–0.801)	5.32E−06	0.664 (0.586–0.752)	1.33E−10
*rs12211410*	6	32157401	T	C	*TNXB*	0.651 (0.540–0.784)	6.35E−06	0.719 (0.619–0.834)	1.56E−05	0.692 (0.615–0.777)	5.78E−10
*rs13199524*	6	32174743	T	C	*TNXB*	0.639 (0.528–0.773)	3.81E−06	0.63 (0.514–0.772)	8.96E−06	0.641 (0.564–0.728)	8.01E−12
*rs12153855*	6	32182782	T	C	*CREBL1*	1.541 (1.296–1.832)	9.46E−07	1.665 (1.353–2.05)	1.46E−06	1.581 (1.405–1.779)	2.96E−14
*rs7130588*	11	75948331	A	G	*C11orf30*	0.770 (0.694–0.853)	5.81E−07	0.794 (0.708–0.889)	7.44E−05	0.778 (0.727–0.833)	4.49E−13
*rs2155219*	11	75976842	T	G	*C11orf30/ LRRC32*	1.360 (1.225–1.510)	8.17E−09	1.319 (1.205–1.444)	1.66E−09	1.323 (1.224–1.429)	1.61E−12

Genome build hg18, dbSNP130. The Filaggrin (*FLG*) locus is not included in the table as replication was not sought.

^a^Meta-analysis of German and UK screening cohorts.

^b^Meta-analysis of replication samples using a random-effects model.

^c^Meta-analysis of the screen and the three replication cohorts using a random-effects model.

### Chromosome 6p21—the MHC locus

We identified a strong signal in the major histocompatibility complex (MHC) region on chromosome 6 with a number of weakly correlated (*r*^2^ < 0.4) markers reaching genome-wide significance. Nine of the 24 SNPs achieved significance in a meta-analysis of the replication cohorts following a correction for multiple testing. Of these, seven SNPs achieved a *P*-value of <5.0E−08 when tested for association in the combined discovery and replication datasets. A peak association *P*-value of 2.96E−14 was achieved at marker *rs12153855* in the class III region of the MHC. This SNP was intronic to the gene *Tenascin XB* (*TNXB;* a gene previously implicated in systemic lupus erythematosus (25)), and positioned just 83 kb from the previously published peak of association for AD within the MHC (rs176095) (26). *Rs176095* achieved nominal significance in our dataset (Table 4, *P* = 0.0031). Independence among these signals was assessed by conditional regression. When conditioned on *rs176095*, the effect of *rs12153855* was weakened, but not eliminated (*β* = 0.3741, SE = 0.0980 and *P* = 0.0001354).

**Table 3.** Association of imputed HLA alleles

*HLA allele*	freq case/cont	OR	95%CI			*P*	Condition on		UK and Ireland						Germany					
							DRB_0701	DRB_0701 + B_4402	Freq case /cont	OR	95%CI			*P*	freq case /cont	OR	95%CI			*P*
HLADRB_0701	0.113/0.152	0.65	0.55	–	0.76	1.36E−07	–	–	0.132/0.153	0.71	0.58	–	0.87	8.29E−04	0.088/0.146	0.54	0.40	–	0.72	2.30E−05
HLADQA_0201	0.126/0.163	0.70	0.59	–	0.81	6.03E−06	0.137	0.106	0.145/0.165	0.75	0.62	–	0.91	4.16E−03	0.102/0.154	0.60	0.46	–	0.79	2.96E−04
HLADQB_0301	0.246/0.193	1.33	1.16	–	1.52	3.82E−05	5.21E−03	0.033	0.234/0.191	1.34	1.12	–	1.59	9.62E−04	0.261/0.206	1.33	1.07	–	1.67	1.21E−02
HLAB_5701	0.025/0.045	0.53	0.39	–	0.73	6.08E−05	9.25E−03	0.012	0.029/0.045	0.55	0.38	–	0.81	2.06E−03	0.019/0.042	0.48	0.27	–	0.83	8.28E−03
HLAB_4402	0.137/0.103	1.39	1.18	–	1.64	9.60E−05	1.52E−03	–	0.166/0.11	1.32	1.09	–	1.60	5.14E−03	0.1/0.059	1.71	1.21	–	2.43	2.33E−03
HLADQB_0202	0.069/0.091	0.67	0.54	–	0.82	1.44E−04	0.622	0.574	0.083/0.092	0.77	0.59	–	0.99	3.97E−02	0.051/0.088	0.53	0.36	–	0.77	8.08E−04
HLADRB_0401	0.113/0.099	1.40	1.15	–	1.70	7.62E−04	0.015	0.104	0.132/0.109	1.27	1.01	–	1.60	3.94E−02	0.089/0.048	1.92	1.26	–	2.93	2.42E−03
HLADQA_0301	0.2/0.187	1.26	1.10	–	1.45	1.26E−03	0.041	0.113	0.236/0.198	1.29	1.09	–	1.52	3.67E−03	0.152/0.122	1.22	0.93	–	1.60	1.50E−01
HLADRB_1101	0.068/0.043	1.52	1.18	–	1.95	1.35E−03	0.010	0.014	0.043/0.037	1.71	1.18	–	2.49	5.02E−03	0.101/0.078	1.37	0.97	–	1.96	7.68E−02

**Table 4.** Association with published GWA markers for AD

Reference	Marker	Risk allele	Gene	Allele 1	Allele 2	AD all					AD and asthma				AD not asthma			
						Effect	StdErr	*P*	Direction	OR	Effect	StdErr	*P*		Effect	StdErr	*P*	
Palmer *et al*. (11)	*FLG* R501X	T	*FLG*	C	T	−1.409	0.236	2.38E−09	−−	0.244	−1.505	0.260	7.21E−09	−−	−1.195	0.272	1.09E−05	−−
Palmer *et al*. (11)	*FLG* *2282del4*	DEL	*FLG*		DEL	−1.720	0.209	1.88E−16	−−	0.179	−1.725	0.237	2.96E−13	−−	−1.673	0.242	4.72E−12	−−
Esparza-Gordillo *et al*. (20)	*rs6661961*	A	*FLG*	T	C	0.338	0.052	8.67E−11	++	1.402	0.386	0.072	6.87E−08	++	0.290	0.063	5.03E−06	++
Esparza-Gordillo *et al*. (20)	*rs877776*	C	*HRNR* near *FLG*	C	G	−0.078	0.076	3.06E−01	−+	0.925	−0.216	0.110	5.00E−02	−−	−0.008	0.092	9.28E−01	−+
Esparza-Gordillo *et al*. (20)	*rs7927894*	A	*c11orf30*	T	C	0.262	0.055	1.73E−06	++	1.300	0.279	0.075	2.10E−04	++	0.261	0.067	9.80E−05	++
Sun *et al*. (19)	*rs3126085*	A	*FLG*	A	G	−0.060	0.079	4.46E−01	−+	0.941	−0.220	0.116	5.65E−02	−−	0.016	0.095	8.67E−01	−+
Sun *et al*. (19)	*rs7701890*	G	*TMEM232-SLC25A46*	A	G	−0.210	0.097	3.06E−02	−−	0.810	−0.196	0.130	1.33E−01	−+	−0.221	0.120	6.65E−02	−−
Sun *et al*. (19)	*rs6010620*	G	*TNFRSF6B-ZGPAT*	A	G	−0.206	0.063	1.18E−03	−+	0.814	−0.248	0.090	5.58E−03	−−	−0.177	0.077	2.10E−02	−+
EAGLE (18)	*rs9050*		*TCHH* near *FLG*	A	C	0.532	0.085	3.91E−10	++	1.703	0.567	0.112	4.30E−07	++	0.478	0.104	4.00E−06	++
EAGLE (18)	*rs479844*	G	*OVOL1*	A	G	−0.220	0.052	2.77E−05	−−	0.803	−0.239	0.073	1.13E−03	−−	−0.187	0.064	3.73E−03	−−
EAGLE (18)	*rs2164983*	A	*ACTL9*	A	C	0.104	0.073	1.53E−01	++	1.110	0.126	0.100	2.06E−01	++	0.116	0.090	1.96E−01	++
EAGLE (18)	*rs2897442*	C	*KIF3A*	T	C	−0.218	0.057	1.18E−04	−−	0.804	−0.250	0.078	1.33E−03	−−	−0.193	0.070	5.97E−03	−−
Hirota *et al*. (21)	*rs13015714*	G	*IL1RL1-IL18R1-IL18RAP*	T	G	−0.116	0.060	5.29E−02	−−	0.887	−0.089	0.082	2.83E−01	−−	−0.126	0.073	8.34E−02	−+
Hirota *et al*. (21)	*rs176095*	A	*GPSM3* (MHC region)	A	G	0.211	0.071	3.13E−03	++	1.234	0.115	0.097	2.37E−01	−+	0.311	0.089	4.63E−04	++
Hirota *et al*. (21)	*rs878860*	C	*OR10A3-NLRP10*	T	C	0.038	0.052	4.67E−01	++	1.041	−0.019	0.073	7.95E−01	−+	0.085	0.063	1.78E−01	++
Hirota *et al*. (21)	*rs6780220*	C	*GLB1*	A	C	−0.157	0.072	2.98E−02	−−	0.852	−0.162	0.100	1.05E−01	−−	−0.165	0.088	6.22E−02	−−
Hirota *et al*. (21)	*rs12634229*	C	*CCDC80*	T	C	−0.057	0.136	6.73E−01	−−	0.942	−0.032	0.188	8.64E−01	−+	−0.076	0.168	6.52E−01	−−
Hirota *et al*. (21)	*rs4722404*	C	*CARD11*	T	C	−0.074	0.055	1.84E−01	−−	0.932	−0.112	0.077	1.47E−01	−−	−0.048	0.067	4.78E−01	−+
Hirota *et al*. (21)	*rs10995251*	C	*ZNF365*	T	C	−0.023	0.054	6.68E−01	−+	0.980	−0.121	0.075	1.08E−01	−−	0.041	0.067	5.40E−01	−+
Hirota *et al*. (21)	*rs16999165*	A	*CYP24A1-PFDN4*	A	G	−0.167	0.142	2.38E−01	−−	0.844	0.004	0.206	9.84E−01	−+	−0.309	0.169	6.76E−02	−−

Hirota study is for Asian (Japanese) samples.

The MHC is characterized by high levels of duplication, polymorphism and LD. We applied two strategies in order to extract maximal information from the observed association.

**Figure 4.**
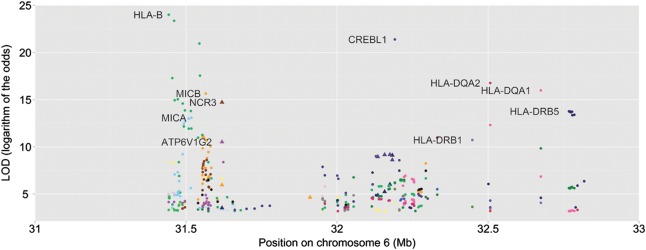
eQTL analysis of the chromosome 6 locus. Transcript abundance associations significant at a 1% false discovery rate. Individual single nucleotide polymorphism (SNP)–transcript associations with a LOD (Logarithm of the Odds) score of >10 are labelled. SNPs are coloured according to their associated transcripts. SNPs found to be significantly associated with AD in the discovery and replication cohorts are shown as triangles. All other SNPs are shown as circles.

First, we used existing expression quantitative trait locus (eQTL) data derived from 496 subjects from the UK portion of the discovery cohort (27) in order to search for eQTL coincident with the seven replicated SNPs. These data were generated using RNA prepared from Epstein-Barr virus (EBV)–transformed lymphoblastoid cell lines followed by expression analysis with Illumina Human 6 BeadChips (27). The proximal genomic region (31–33 Mb) encapsulating the seven replicated SNPs was examined for *cis*- or *trans*-acting eQTL capable of affecting any transcript represented on the Illumina 6 Human BeadChip (including >33 000 RefSeq transcripts). The seven SNPs of interest were not found to align with the most significant eQTL in the region, although effects in other cell types or physiological conditions cannot be excluded. The SNPs nevertheless did show moderate levels of association with a number of gene expression traits (Fig. 4). The most significant hits were the natural cytotoxicity triggering receptor *NCR3* (*rs2844509*, LOD = 14.7), ATPase *ATP6V1G2* (*rs2844509*, LOD = 10.5), activating transcription factor *CREBL1* (four SNPs, LOD range = 9.2–8.6), and *HLA-DRB5* (five SNPs, LOD range = 9.1–3.5).

We next imputed classical HLA alleles using the dense SNP genotype data of the discovery phase subject set, using a published statistical methodology previously shown to impute HLA alleles from SNP data with 92–98% accuracy at the four-digit level. (28–30). Significant associations were observed for HLA-B*5701 (*P* = 6.08E−05), B*4402 (*P* = 9.60E−05), DRB1*0701 (*P* = 1.36E−07), DRB1*0401 (*P* = 7.62E−04), DRB1*1101 (*P* = 1.35E−03), DQA1*0201 (*P* = 6.03E−06), DQA1*0301 (*P* = 1.26E−03), DQB1*0301 (*P* = 3.82E−05) and DQB1*0202 (*P* = 1.44E−04). Taking the most significant association, HLA-DRB1*0701 (protective for AD OR = 0.65) and using conditional analysis, we tested for independence of the association from the other HLA loci. Only a marginal association of HLA-B*4402 remained (*P* = 0.0015). When conditioning on both HLA-DRB1*0701 and HLA-B*4402, none of the other loci were significant (*P* < 0.01) after correction for multiple testing. These results indicate that the association signal observed within the margins of the MHC may reflect the action of classical HLA alleles with typed markers acting as proxies. In particular, our data suggest that HLA-DRB1*0701 confers a significant protective effect on AD, and that HLA-B*4402 slightly increases the risk of AD independent of DRB1*0701 (Table 3).

Polymorphisms in the DR beta chains of the Class II HLA molecule specify peptide-binding affinities. The HLA-DRB1*0701 allele found here to associate with AD has previously been associated with a range of T-cell mediated inflammatory diseases including podoconiosis (31) (an inflammatory disease of the lower legs that occurs after long-term skin exposure to mineral microparticles), psoriasis (32–34) and vitiligo (35). In each of these disorders, the HLA-DRB1*0701 allele increases the disease risk, suggesting that it may have particular relevance in response to cutaneously exposed antigens.

We found that the locus did not have an effect on the AD plus asthma phenotype. Of the 24 SNPs that attained a *P*-value of <1.0E−05 for AD in the discovery phase, 11 achieved *P*-values below this threshold when the case status was limited to individuals without asthma compared with only two SNPs in cases with asthma (Supplementary Material, Table S1). In addition, the signal reported here did not coincide with that identified in a large-scale GWAS of asthma (36), where the asthma association was positioned in the HLA-DQ region (*rs9273349*), and showed low levels of correlation (*r*^2^ < 0.2) with the markers found to associate with AD in this dataset.

### Chromosome 5q13

We identified a second major site of association on chromosome 5q31. Eight SNPs in this region achieved *P* < 1.0E−05 for AD in the discovery phase. In contrast with the MHC, half of these markers (four out of eight) had *P-*values below this threshold when the case status was limited to the ‘AD plus asthma’ subgroup, whereas only one SNP retained significance in the ‘AD no asthma’ subgroup (Supplementary Material, Table S3). These data suggest that the chromosome 5 locus may exert overlapping effects, affecting both AD and asthma. Consistent with these observations, markers in this region (spanning between the *RAD50* locus to the *IL13* locus) have previously been implicated in the aetiology of asthma by GWA (36). They have also shown genome-wide association with the intermediate phenotypes of serum IgE and eosinophil numbers (37,38).

Five out of eight discovery phase SNPs retained significance in the replication data following correction for multiple testing. All five SNPs achieved a *P*-value of <5.0E−08 in the combined discovery and replication datasets (with a peak association *P*-value of 1.65E−17 at *rs1295686*). Two distinct, but incompletely independent, association signals were observed with one block containing three of the SNPs *rs2897433*, *rs6871536*, *rs2158177* and the other block containing *rs1295686* and *rs20541* (LD, *r*^2^ > 0.89 within the two blocks, HapMap III, release 2 CEU + TSI), suggesting that there may be more than one susceptibility locus within the region.

One of the most strongly associated SNPs (*rs20541*) was a missense variant in the gene encoding Interleukin 13 (*IL13* + 2044G > A); a cytokine predominantly secreted by Th2 cells and an important factor in the regulation of allergic inflammation. This variant has been repeatedly associated with asthma as well as being shown to result in a distinct isoform of IL-13 with increased biological activity (39).

A previous GWAS of AD has identified a peak marker (*rs2897442*) located in an intron of *KIF3A* in the 5q31 genomic region (18) This signal is located >50 kb away from the site of association reported here, yielded *P*-values of lower significance (Table 4), and demonstrates only minimal levels of LD (*r*^2^ < 0.1, *D*’ < 0.4 based on the CEU 1000 Genomes dataset) with the five replicated SNPs in this current study. Subsequent high-resolution mapping of the region, however, revealed a second independent signal (*rs848*), in only weak LD with *rs2897442*, positioned in IL13 and showing strong LD with the functional IL13 variant found to be associated here (*rs20541*). These results are consistent with the data that we present here, indicating that more than one effect may reside within this region.

### Chromosome 11q13.5

We mapped a third genome-wide significant signal to an intergenic interval on chromosome 11q13.5 between the uncharacterized protein coding gene *C11orf30* and the gene encoding the leucine-rich type I membrane protein, *LRRC32*.This region has previously been associated with AD (20). Two markers at this site (*rs7130588* and *rs2155219*) demonstrated strong and consistent patterns of association with AD in the discovery and replication panels (Table 2). *P*-values did not appear to differ systematically between the ‘AD plus asthma’ and the ‘AD no asthma’ strata of the analysis. Examination of the HapMap CEU and TSI datasets revealed high levels of LD between marker *rs7130588* and the *rs7927894* SNP previously shown to be associated with AD (20) (*r*^2^ = 0.91, HapMap III, release 2), indicating that these SNPs are likely to represent a single risk locus.

### Analysis of previously identified risk alleles for atopic disease and psoriasis

A composite list of variants associated with atopic diseases [asthma, AD and allergic rhinitis (AR)], and the chronic inflammatory skin disease psoriasis, by means of GWAS, was compiled from the catalogue of Published GWAS (40) and associated reference lists. Where possible, we examined these variants for association with AD in our discovery dataset, either directly or by imputation.

Consistent with historical evidence of genomic correspondence (1), 13 variants previously shown to associate with psoriasis by GWAS achieved low *P*-values in our dataset (Table 5). Approximately two-thirds of these variants showed opposite risk profiles for AD versus psoriasis. The remaining one-third exhibited an association with the same allele. These patterns may reflect genetic contributions towards the divergent and shared features of these two common dermatological diseases. A propensity towards opposing risk profiles is consistent with the infrequency of the two diseases' co-occurrence (41).

**Table 5.** Association with published GWA markers for psoriasis

Reference	SNP	Risk allele	Gene	Allele 1	Allele 2	Effect	StdErr	*P*	OR	Direction
Tsoi *et al.* (47)	*rs11121129*	A	*SLC45A1,TNFRSF9*	A	*G*	0.1275	0.0561	2.30E−02	1.14	Same
Tsoi *et al.* (47)	*rs7552167*	*G*	*IL28RA*	A	*G*	0.1531	0.0732	3.65E−02	1.17	Opposite
Tsoi *et al.* (47)	*rs7536201*	C	*RUNX3*	A	*G*	0.0769	0.0559	1.69E−01	1.08	
Nair *et al.* (73)	*rs2201841*	*G*	*IL23R*	A	*G*	−0.0216	0.0556	6.98E−01	0.98	
Tsoi *et al.* (47)	*rs9988642*	T	*IL23R*	T	*C*	0.0783	0.0621	2.07E−01	1.08	
Zhang *et al.* (74)	rs4085613	?	*LCE3D*	T	*G*	0.0048	0.0546	9.30E−01	1.00	
WTCCC2 (75)	*rs4112788*	?	*LCE3D*	A	*G*	0.0041	0.0539	9.40E−01	1.00	
Tsoi *et al.* (47)	*rs6677595*	T	*LCE3B,LCE3D*	T	*C*	−0.0037	0.0539	9.45E−01	1.00	
WTCCC2 (75)	*rs702873*	*G*	*REL*	T	*C*	−0.0890	0.0522	8.85E−02	0.91	
Tsoi *et al.* (47)	*rs62149416*	T	*FLJ16341,REL*	A	*C*	−0.0464	0.0523	3.75E−01	0.95	
Tsoi *et al.* (47)	*rs10865331*	A	*B3GNT2*	A	*G*	−0.0434	0.0528	4.11E−01	0.96	
WTCCC2 (75)	*rs17716942*	A	*IFIH1*	T	*C*	−0.0334	0.0852	6.95E−01	0.97	
Tsoi *et al.* (47)	*rs17716942*	T	*KCNH7,IFIH1*	T	*C*	−0.0334	0.0852	6.95E−01	0.97	
WTCCC2 (75)	*rs27524*	A	*ERAP1*	A	*G*	−0.0230	0.0532	6.66E−01	0.98	
Tsoi *et al.* (47)	*rs27432*	A	*ERAP1*	A	*G*	0.0002	0.0574	9.97E−01	1.00	
Nair *et al.* (73)	*rs20541*	*G*	*IL13*	A	*G*	0.3547	0.0630	1.79E−08	1.43	Opposite
Tsoi *et al.* (47)	*rs1295685*	*G*	*IL13,IL4*	A	*G*	0.3782	0.0647	4.94E−09	1.46	Opposite
Tsoi *et al.* (47)	*rs2233278*^*a*^	*C*	*TNIP1*	T	*C*	−0.0057	0.1027	9.55E−01	0.99	
Nair *et al.* (73)	*rs17728338*	A	*TNIP1*	A	*G*	−0.1376	0.1221	2.60E−01	0.87	
Nair *et al.* (73)	*rs2082412*	*G*	*IL12B*	A	*G*	0.1481	0.0639	2.05E−02	1.16	Opposite
Cargill *et al.* (76)	*rs3212227*	A	*IL12B*	T	*G*	−0.1525	0.0639	1.70E−02	0.86	Opposite
Zhang *et al.* (74)	*rs3213094*	?	*IL12B*	T	*C*	0.1565	0.0639	1.43E−02	1.17	
WTCCC2 (75)	*rs3213094*	?								
Ellinghaus *et al.* (77)	*rs2546890*	A	*IL12B*	A	*G*	0.0075	0.0521	8.86E−01	1.01	
Hüffmeier *et al.* (78)	*rs12188300*	*T*	*IL12B*	A	*T*	0.2009	0.1092	6.57E−02	1.22	
Tsoi *et al.* (47)	*rs12188300*^a^	*T*	*IL12B*	A	*T*	0.2009	0.1092	6.57E−02	1.22	
Tsoi *et al.* (47)	*rs9504361*	A	*EXOC2,IRF4*	A	*G*	0.0234	0.0526	6.56E−01	1.02	
Russell *et al.* (79)	*HLA-C*	^a^0602		^a^0602	Others	−0.2695	0.0946	4.40E−03	0.76	Opposite
Tsoi *et al.* (47)	*rs4406273*	A	*HLA-B,HLA-C*	C	*G*	−0.0251	0.1367	8.55E−01	0.98	
WTCCC2 (75)	*rs240993*	A	*TRAF3IP2*	T	*C*	0.0586	0.0585	3.17E−01	1.06	
Hüffmeier *et al.* (78)	*rs13190932*	A	*TRAF3IP2*	A	*G*	0.1121	0.1156	3.32E−01	1.12	
Tsoi *et al.* (47)	rs33980500	*T*	*TRAF3IP2*	A	*G*	−0.3152	0.1461	3.10E−02	0.73	Opposite
Ellinghaus *et al.* (77)	*rs13210247*	*G*	*TRAF3IP2*	A	*G*	−0.1049	0.1086	3.34E−01	0.90	
Tsoi *et al.* (47)	*rs582757*	*C*	*TNFAIP3*	T	*C*	−0.1176	0.0574	4.05E−02	0.89	Same
Nair *et al.* (73)	*rs610604*	*G*	*TNFAIP3*	T	*G*	−0.0889	0.0548	1.05E−01	0.91	
Tsoi *et al.* (47)	*rs2451258*	*C*	*TAGAP*	T	*C*	−0.0813	0.0537	1.30E−01	0.92	
Tsoi *et al.* (47)	*rs2700987*	A	*ELMO1*	T	*C*	0.0665	0.0538	2.17E−01	1.07	
Tsoi *et al.* (47)	*rs11795343*	*T*	*DDX58*	T	*C*	0.0521	0.0521	3.17E−01	1.05	
Tsoi *et al.* (47)	*rs10979182*	A	*KLF4*	A	*G*	−0.0089	0.0541	8.69E−01	0.99	
Tsoi *et al.* (47)	*rs1250546*	A	*ZMIZ1*	A	*G*	−0.1066	0.0557	5.58E−02	0.90	
Tsoi *et al.* (47)	*rs645078*	A	*RPS6KA4,PRDX5*	A	*C*	0.0045	0.0533	9.33E−01	1.00	
Tsoi *et al.* (47)	*rs4561177*	A	*ZC3H12C*	A	*G*	0.0652	0.0521	2.11E−01	1.07	
Tsoi *et al.* (47)	*rs3802826*	A	*ETS1*	A	*G*	0.0849	0.0781	2.77E−01	1.09	
Nair *et al.* (73)	*rs2066808*	A	*IL23A-STAT2*	A	*G*	0.1455	0.0950	1.26E−01	1.16	
Tsoi *et al.* (47)	*rs2066819*	*C*	*STAT2,IL23A*	T	*C*	−0.1435	0.1008	1.54E−01	0.87	
Stuart *et al.* (80)	*rs12586317*	*T*	*NFKBIA*							
WTCCC2 (75)	*rs8016947*	*C*	*NFKBIA*	T	*G*	0.0701	0.0511	1.70E−01	1.07	
Tsoi *et al.* (47)	*rs8016947*	*G*	*NFKBIA*	T	*G*	0.0701	0.0511	1.70E−01	1.07	
Tsoi *et al.* (47)	*rs367569*	*C*	*PRM3,SOCS1*	T	*C*	−0.1241	0.0584	3.35E−02	0.88	Same
Stuart *et al.* (80)	*rs10782001*	*G*	*FBXL19*	A	*G*	−0.0311	0.0541	5.65E−01	0.97	
Tsoi *et al.* (47)	*rs12445568*	*C*	*PRSS53,FBXL19*	T	*C*	−0.0503	0.0533	3.46E−01	0.95	
Stuart *et al.* (80)	*rs4795067*	*G*	*NOS2*	A	*G*	−0.0063	0.0630	9.20E−01	0.99	
Tsoi *et al.* (47)	*rs28998802*	A	*NOS2*	C	*G*	−0.1283	0.1265	3.10E−01	0.88	
Tsoi *et al.* (47)	*rs963986*^a^	*C*	*PTRF,STAT3,STAT5A/B*	C	*G*	0.0829	0.0711	2.44E−01	1.09	
Tsoi *et al.* (47)	*rs11652075*	*C*	*CARD14*	T	*C*	−0.1287	0.0511	1.19E−02	0.88	Same
Tsoi *et al.* (47)	*rs545979*	*T*	*POL1,STARD6,MBD2*	T	*C*	−0.1628	0.0576	4.75E−03	0.85	Opposite
Tsoi *et al.* (47)	*rs34536443^a^*	*G*	*TYK2*	T	*C*	0.0090	0.1030	9.30E−01	1.01	
WTCCC2 (75)	*rs12720356*	A	*TYK2*	A	*C*	0.0341	0.0870	6.95E−01	1.03	
Tsoi *et al.* (47)	*rs892085*	A	*ILF3,CARM1*	A	*G*	−0.0083	0.0538	8.77E−01	0.99	
Capon *et al.* (81)	*rs495337*	?	*ZNF313*	A	*G*	−0.0602	0.0521	2.47E−01	0.94	
Tsoi *et al.* (47)	*rs1056198*	*C*	*RNF114*	T	C	−0.0576	0.0519	2.67E−01	0.94	
Tsoi *et al.* (47)	*rs4821124*	*C*	*UBE2L3*	T	C	−0.0141	0.0662	8.32E−01	0.99	

Zhang study is for Asian samples (Chinese).

^a^rs2233278 C/G rs12188300 A/T rs34536443 G/C rs963986 C/G.

Two SNPs attained significance at a genome-wide level. Both exhibited opposing effects in AD and psoriasis, and localized to the gene *IL13* (*rs1295685*, *P* = 4.94E−09 and *rs20541*, *P* = 1.786E−08). *IL13* is an immunoregulatory cytokine primarily secreted by activated T helper type II (Th2) cells, triggering B lymphocytes to switch towards IgE production. An inverse association at this site may therefore contribute towards the distinct immune profiles of psoriasis and AD (type I and type II, respectively). Similarly, suggestive opposing signals were also seen at several markers positioned in and around the cytokine interleukin 12B (*IL12B*), a key cytokine in Th1 lineage development and survival (42–44). AD and psoriasis also differ in their susceptibility towards cutaneous bacterial and viral infection (41). Consistent with this, suggestive opposing signals were seen at a single marker proximal to interleukin 28 receptor alpha (interferon, lambda receptor, *IL28RA*); a gene previously implicated in viral infection and resolution (45), and at a missense mutation in *TRAF3IP2*; an evolutionarily conserved component of the host antiviral signalling pathway (46).

Four variants showed equivalent risk profiles in AD and psoriasis, such that both diseases associated with the same alleles. These included a common, damaging missense variant (47,48) in the gene *CARD14*. CARD14 is predominantly expressed in the skin, where it activates the NF-kB pathway augmenting the keratinocyte response to inflammatory cytokines.

A single variant located in the *c11orf30*-*LRRC2* region has previously been shown to associate with AR by GWAS (*rs2155219*) (49). This variant yielded genome-wide significant evidence of association with AD in our discovery phase (AD *P* = 8.17E−09, OR 1.36); an effect of equivalent direction to that previously reported in AR, and of similar significance in AD patients with (*P* = 3.67E−06) and without (*P* = 6.01E−06) asthma (Table 6).

**Table 6.** Association with published GWA markers for AR

Reference	SNP	Risk allele	Gene	Allele 1	Allele 2	AD					AD and asthma				AD not asthma			
						Effect	StdErr	*P*	OR	Direction	Effect	StdErr	*P*		Effect	StdErr	*P*	
Ramasamy *et al.* (49)	*rs2155219*	T	*c11orf30-LRRC2*	T	G	0.3076	0.0534	**8.17E**−**09**	1.36	same	0.3454	0.0746	**3.67E**−**06**	++	0.2958	0.0653	**6.01E**−**06**	++

We also tested in our dataset 21 variants previously implicated in the aetiology of asthma by GWA. While none of these achieved criteria for genome-wide significance, suggestive signals were observed on chromosome 11 around the *c11orf30*-*LRRC2* locus described above for its role in AR (AD *P* = 5.81E−07, *rs7130588*). The effect at this site matched the direction of that reported for asthma, suggesting that this region may harbour mechanisms capable of moderating multiple atopic disease processes.

Lower magnitude signals were observed at a number of other positions, including the chromosome 17 *GSDMB*-*ORMDL3*-*GSDMA* locus, *SMAD3*, the interleukin 6 receptor (*IL6R*), *FLJ44477*-*USP38* and *SUOX*-*IKZF4* (Table 7). These all demonstrated a common pattern of association, achieving the most significant *P*-values in ‘AD plus asthma’ cases and showing a complete absence of association in ‘AD no asthma’ cases. This implies that association at these sites may be primarily driven by asthma status.

**Table 7.** Association with published GWA markers for asthma

Reference	SNP	Risk allele	Gene	Allele 1	Allele 2	AD					AD and asthma				AD not asthma			
						Effect	StdErr	*P*	OR	Direction	Effect	StdErr	*P*		Effect	StdErr	*P*	
Moffatt *et al*. (67)	*rs7216389*	T	*ORMDL3*	T	C	0.0882	0.0506	8.12E−02	1.09	Same	0.2222	0.0701	**1.53E**−**03**	++	0.0056	0.0617	9.28E−01	−+
Himes *et al*. (68)	*rs1588265*	C	*PDE4D*	−−	−−	−−	−−	−−										
Sleiman *et al*. (69)	*rs2786098*	C	*DENND1B*	T	G	−0.0912	0.063	1.48E−01	0.91	Same	−0.0116	0.0868	8.94E−01	−+	−0.1543	0.0783	**4.88E**−**02**	−−
GABRIEL (36)	*rs3771166*	A	*IL1RL1-IL18R1*	A	G	−0.1557	0.0539	**3.88E**−**03**	0.86	Same	−0.1148	0.0755	1.28E−01	−−	−0.1783	0.0666	**7.38E**−**03**	−−
GABRIEL (36)	*rs9273349*	C	*MHC*	−−	−−	−−	−−	−−										
GABRIEL (36)	*rs1342326*	C	*IL33*	A	C	−0.07	0.0677	3.02E−01	0.93	Same	−0.0783	0.0935	4.02E−01	−−	−0.073	0.0831	3.79E−01	−−
GABRIEL (36)	*rs744910*	A	*SMAD3*	A	G	−0.1045	0.0515	**4.24E**−**02**	0.90	Same	−0.1686	0.0709	**1.75E**−**02**	−−	−0.0727	0.0627	2.46E−01	−−
GABRIEL (36)	*rs2305480*	A	*GSDMB*	A	G	−0.1441	0.0507	**4.52E**−**03**	0.87	Same	−0.2873	0.0714	**5.79E**−**05**		−0.0652	0.0617	2.91E−01	
GABRIEL (36)	*rs3894194*	A	*GSDMA*	A	G	0.1453	0.0513	**4.64E**−**03**	1.16	Same	0.2761	0.0708	**9.60E**−**05**		0.0596	0.0629	3.44E−01	
GABRIEL (36)	*rs2284033*	A	*IL2RB*	A	G	−0.0235	0.0519	6.51E−01	0.98	Same	0.0715	0.072	3.21E−01	++	−0.0802	0.0641	2.11E−01	−−
Noguchi (70)	*rs3019885*	G	*SLC30A8*	T	G	−0.1174	0.0524	**2.51E**−**02**	0.89	Same	−0.1354	0.072	6.01E−02	−+	−0.1225	0.0653	6.08E−02	−−
Noguchi (70)	*rs987870*	C	*HLA*−*DP*	A	G	−0.0375	0.0744	6.14E−01	0.96	Same	−0.0676	0.1025	5.10E−01	−−	−0.022	0.0915	8.10E−01	+−
EVE (71)	*rs11078927*	C	*GSDMB*	T	C	−0.1436	0.0507	**4.65E**−**03**	0.87	Same	−0.2883	0.0714	**5.44E**−**05**	−−	−0.0629	0.0617	3.08E−01	−−
EVE (71)	*rs1837253*	C	*TSLP*	T	C	0.0139	0.061	8.20E−01	1.01		−0.0746	0.086	3.86E−01	−−	0.0805	0.0747	2.81E−01	++
EVE (71)	*rs10173081*	C	*IL1RL1*	T	C	−0.0723	0.075	3.35E−01	0.93		−0.0349	0.1038	7.37E−01	+−	−0.0942	0.0927	3.09E−01	−−
EVE (71)	*rs1101999*		*PYHIN1*															
Hirota *et al*. (26)	*rs7686660*	T	*FLJ44477 - USP38*	T	G	0.0451	0.0589	4.44E−01	1.05	Same	0.1762	0.0839	**3.57E**−**02**	++	−0.036	0.0711	6.12E−01	−+
Hirota *et al*. (26)	*rs10508372*	C	*KRT8P16 - TCEB1P3*	A	G	0.0435	0.1058	6.81E−01	1.04	Opposite	−0.069	0.1488	6.43E−01	−−	0.1279	0.128	3.18E−01	++
Hirota *et al*. (26)	*rs1701704*	G	*SUOX - IKZF4*	T	G	−0.0979	0.0541	7.02E−02	0.91	Same	−0.1547	0.0746	**3.81E**−**02**	−−	−0.0545	0.0666	4.13E−01	−−
Ferreira *et al*. (72)	*rs4129267*	T	*IL6R*	T	C	0.122	0.0513	**1.74E**−**02**	1.13	Same	0.1904	0.0713	**7.56E**−**03**	++	0.078	0.0637	2.20E−01	++
Ferreira *et al*. (72)	*rs7130588*	G	*c11orf30-LRRC2*	A	G	−0.2619	0.0524	**5.81E**−**07**	0.77	Same	−0.2733	0.0727	**1.71E**−**04**	−−	−0.2647	0.0644	**3.94E**−**05**	−−

Noguchi and Hirota studies are for Asian (Japanese) samples, and the EVE signal for *PYHIN1* was detected only in individuals of African descent. *P*-values below a nominal threshold of 0.05 are highlighted in bold.

## DISCUSSION

We have presented here a GWAS of AD in a large panel of Northern European cases with childhood onset disease and known asthma status. We identified loci involved in the aetiology of AD at the well-established *FLG* locus on chromosome 1, the MHC on chromosome 6p21, the *RAD50/IL13* locus on chromosome 5q31 and an 11q13.5 locus in strong LD with a previously characterized AD susceptibility site (20). We show that variation exists between these loci in terms of their contribution towards co-morbid asthma, and furthermore, that genomic overlap between AD and psoriasis can be explained by a pattern of systematic association with the same markers, but not necessarily the same alleles.

The MHC has only previously been implicated in AD in a single cohort of Japanese origin (21). Here, we observe an independent association signal in close proximity with that previously reported. The associated variants did not exhibit pronounced direct effects on gene expression in the cell type studied, indicating an alternative mode of action, indirect association or tissue-specific effects. By imputation, we are able to show that this signal, identified here in a Caucasian population of Northern European origin, is driven by independent effects of two classical HLA alleles, HLA-DRB*0701 and HLA-B*4402.

The MHC complex exhibits high levels of population divergence (50,51) reflecting, among other factors, local adaptation to parasite and pathogen communities. The region demonstrates considerable geographical variation in LD (52) and allele frequencies, as well as regional differences in the allelic spectrum (53), implying the presence of distinct core haplotypes. It remains unclear the extent to which the deviation in the patterns of association between Caucasian and Asian populations at this locus can be attributed to differences in the underlying LD structure (and the same causal alleles) or separable, co-localized population-specific effects.

Pleiotropy is a common feature emerging from recent GWAS. Autoimmune diseases in particular have been mapped to many shared loci with variable specificities for different diseases (54). Attempts to model this phenomenon have indicated that while the number of traits affected by any one gene is likely to be small, those genes associated with higher levels of pleiotropy are likely to exert larger effects (55). As such, designation of shared loci may represent a useful metric for the prioritization of genes for further detailed mapping and functional analysis.

Here, by examination of the endophenotypes ‘AD and asthma’ and ‘AD no asthma’ and by comparison of our results with those of existing GWAS for other related phenotypes, we have found that AD is determined by a combination of specific and shared effects, with the *c11orf30*-*LRRC2* locus on chromosome 11 in particular showing effects on AD, asthma and AR. Co-localization between AD, asthma and rhinitis was largely mediated by the same alleles, whereas in the sites of co-localization between AD and psoriasis association was mediated by a combination of shared and opposing alleles, with the most significant effects operating in opposing directions. These data are consistent with observations of clinical co-occurrence between AD, rhinitis and asthma, and relative clinical independence between AD and psoriasis.

While this study is well powered for the detection of small, common effects underpinning the genetic origins of AD, it suffers from several limitations. Asthma status was not available in all of our population-based cohorts, increasing the potential for misclassification bias. This may have resulted in a modest loss of statistical power, given the prevalence of the phenotypes studied. This effect has been mitigated through the use of strict diagnostic criteria for cases status (see Materials and Methods). Inclusion of cohorts from multiple geographic origins may lead to unrecognized population heterogeneity and false-positive errors. We have minimized this influence by removal of non-European individuals; by principal components analysis and inclusion of the top 10 components in the association model and by separate analysis of the UK–Irish and German portions of the cohort, followed by meta-analysis with application of a genomic control estimate.

Until recently, the genetic origins of AD have been characterized by a single robust major locus (*FLG*) and a multitude of small, poorly replicated effects. Our large GWAS has confirmed a number of known effects and demonstrated a marked variation in the phenotypic contribution of particular loci. The data presented here thus support a complex model of AD that incorporates both epidermal barrier and immune mechanisms (56), and a combination of shared and AD-specific effects.

## MATERIALS AND METHODS

### Participants

The discovery dataset comprised 5617 individuals of European ancestry, including 1563 AD cases, and 4054 population-based controls. Individuals with AD were recruited from tertiary dermatology clinics across three centres (Great Ormond Street Hospital, London; Our Lady's Children's Hospital, Dublin; and University Hospital rechts der Isar, München). The diagnosis of AD was made by experienced dermatologists or paediatricians according to the UK diagnostic criteria (57). All AD cases had a reported disease onset in childhood and suffered from moderate-to-severe disease at the time of recruitment. AD-free controls were drawn from three population-based studies; the cross-sectional International Study of Asthma and Allergies in Childhood (ISAAC) Phase II (58), the Avon Longitudinal Study of Children and Parents (ALSPAC) (59) and the 1958 Birth Cohort (60). Asthma status (defined elsewhere (36)) was available for all datasets with the exception of the 1958 birth cohort. The 4,054 population controls were not selected to be asthma-free, in order to allow unbiased analysis of the three endophenotypes; AD, AD and asthma, AD no asthma.

The replication dataset comprised 5446 individuals of European Ancestry, including 2286 AD cases, and 3160 AD-free controls. AD cases were dermatologist-diagnosed, recruited from dermatology clinics at five sites across Germany, Ireland and Sweden. Samples utilized in the replication phase were drawn from the KORA S4/F4 population-based survey (61) ISAAC Phase II, the Swedish longitudinal birth cohort BAMSE (62) and a previously described set of unselected Irish population controls derived from the Trinity College Dublin Biobank (63). Controls of German and Swedish origin were both AD- and asthma-free. Disease status was not available for the Irish Biobank controls.

The study was approved by the institutional review boards at each of the participating study sites. A written informed consent for the collection and genotyping of DNA was obtained from all study participants and/or their parents/guardians where appropriate.

### Genotyping

Genome-wide genotyping was performed on bar-coded LIMS (Laboratory Information Management System) tracked samples using the Illumina Sentrix HumanHap300 Genotyping BeadChip (ALSPAC, UK families, German samples, ISAAC) and the Illumina Human 610-Quad BeadChip (Irish samples, 1958BC) (Illumina, San Diego). BeadChips were processed within an automated BeadLab at the Centre National de Genotypage as per the manufacturer's instructions. Samples were subject to strict quality control criteria including assessment of concentration, fragmentation and response to PCR. A total of 20 µl of DNA aliquoted to a concentration of 50 ng/µl was used for each array. Replication phase genotyping was performed using matrix-assisted laser desorption/ionization time-of flight (MALDI-TOF) mass spectrometry (http://www.sequenom.com), or Applied Biosystems TaqMan probes (http://www.appliedbiosystems.com/). Genotyping of Filaggrin variants R501X, 2282del4, R2447X and S3247X was performed as previously described (12,15,23). *FLG* genotype availability and distributions are given in Supplementary Material, Table S2.

### Statistical analysis

In the discovery phase, genome-wide genotypes were used for controlling the quality of the samples. First individuals with call rates <95% or sex inconsistencies were excluded. Next principal components analysis and identity-by-descent (IBD) estimation were done using EIGENSOFT and PLINK version 1.07, respectively. Duplicates and individuals who were possibly non-European were removed, and from each group of subjects who exhibited a strong relationship only one subject was randomly chosen (full-sibs or parent-offspring). By using this filtered sample set, we calculated quality control statistics, and SNPs with call rates <98% or SNPs with a Hardy–Weinberg equilibrium test *P*–value <1.0E−06 were excluded. Finally, 268 034 experimentally genotyped SNPs with a minor allele frequency >1% were used for imputation analysis. Imputation was done using Mach and minimac software, following the instructions provided by the author (http://genome.sph.umich.edu/wiki/Minimac). HapMap CEU release 22 reference panel was used, and the imputed genetic dosage with *R*-squared statistics >0.3 was used for the association analysis. As a result, 2 543 887 SNPs were imputed, and association tests were performed for 2 406 139 SNPs with an imputation quality metric *R*-squared value of >0.3 and a minor allele frequency of >0.01. Association was tested separately in the UK–Irish and German portions of the cohort using mach2dat software (64,65), incorporating the top 10 principal components as covariates. These data were then combined by fixed-effects meta-analysis after genomic control was applied to both datasets.

Since null mutations in the *FLG* gene are known to be associated with AD, we performed additional regression analysis for all SNPs around the *FLG* gene conditioned on the two most common *FLG* null alleles, R501X and 2282del4.

We also performed imputation of HLA alleles by using HLA*IMP software (29,30). To analyse imputed HLA alleles, we treated post-probabilities as genetic dosages of the alleles and estimated their effects in the regression model in conjunction with principal components as used in genome-wide analysis. The frequencies of HLA alleles were calculated as the sum of genetic dosage divided by the number of chromosomes. The logistic regression analysis was performed by *R* (release 2.13.0) (66). In this analysis, significance levels were Bonferroni-corrected at each test group, i.e. nominal *P*-value of 5.0E−02 divided by the number of tests carried out for one HLA locus. From the results of association analysis, we chose SNPs which showed *P*-values <1.0E−05 and evaluated these signals in independent replication samples. SNPs in LD (*r*^2^ > 0.3 in HapMap_CEU) with another marker with a lower *P*-value, were not included to avoid redundancy. As a result, we chose 47 SNPs for the replication stage. In addition, SNPs categorized as candidate functional SNPs were selected from each LD bin. Association was tested in the replication sets by meta-analysis using a random-effects model as implemented in R (release 2.13.0). In order to control for known AD susceptibility alleles located within the EDC on chromosome 1, significant markers were included in a logistic regression incorporating the two most common *FLG* null alleles.

Genuine replication was considered if *P*-values exceeded the threshold of 1.0E−03 (0.05/47) and consistent effects in the same direction were observed in the GWA as well as all replication cohorts. Where markers implicated in psoriasis by previous GWAS were not genotyped in our dataset, they were imputed by mach1 phasing and minimac imputation using the 1000Gv3 template for EUR population as a reference panel, and employing only those variants within 1 Mb of the target SNP. All *r*^2^ were >0.8, with the exception of *rs4406273* which showed *r*^2^ = 0.67

### eQTL

Genome-wide expression QTL (eQTL) data were available as part of a prior study described in detail previously (27). Briefly, the eQTL data were generated from RNA extracted from EBV-transformed lymphoblastoid cell lines originating from 496 children within the UK portion of the discovery cohort. Transcript abundance was measured on a genome-wide scale using the Illumina Human 6 BeadChip and examined in combination with the 1000 genomes imputed genotype data described above to map genetic factors capable of moderating expression. Non-genetic effects were controlled for using a principal components approach shown to increase power for the detection of *cis* and *trans*-acting effects.

## SUPPLEMENTARY MATERIAL

Supplementary Material is available at *HMG* online.

*Conflicts of Interest statement*. None declared.

## FUNDING

This work was supported by the Wellcome Trust (077326/Z/05/Z), the Medical Research Council (G0700314), the French Ministry of Higher Education and Research, the British Skin Foundation, Génome Québec, le Ministère de l'Enseignement supérieur, de la Recherche, de la Science et de la Technologie (MESRST) Québec and McGill University, the National Eczema Society, the German Federal Ministry of Education and Research (BMBF), the State of Bavaria and the German National Genome Research Network (NGFN-2, NGFNPlus: 01GS0823, 01GS 0818) and donations from anonymous families affected by eczema in the Tayside Region of Scotland. The BAMSE study was supported by the Swedish Research Council, Stockholm County Council, Centre for Allergy Research, Karolinska Institutet and the Swedish Heart Lung Foundation.

S.W. is supported by a Heisenberg Professorship of the DFG (grant WE 2678/7-1), the Christiane Kühne Centre for Allergy Research and Education (http://www.ck-care.ch/), the Graduate School of Information Science in Health of the Technische Universität München (TUM-GSISH), and S.W. and S.J.B. are supported by the COST action ‘SkinBAD’. A.D.I., S.J.B. and G.M.O.'R. are supported by the National Children's Research Centre, Dublin. A.D.I., S.J.B., G.M.O.'R., P.B. and W.H.I.M. are supported by the Wellcome Trust (reference: 090066/B/09/Z, 092530/Z/10/Z). S.W.-O. is supported by the Freemasons Grand Charity. S.J.B. holds a Wellcome Trust Fellowship (ref 086398/Z/08/Z). Funding to pay the Open Access publication charges for this article was provided by the Wellcome Trust.

## Supplementary Material

Supplementary Data
