# Supplementary material for: A genome-wide association study of atopic dermatitis identifies loci with overlapping effects on asthma and psoriasis
Source: Hum Mol Genet. 2013 Jul 25;22(23):4841–56. doi: 10.1093/hmg/ddt317 (PMC3820131; doi:10.1093/hmg/ddt317)
Supplement: Supplementary Data [file supp_22_23_4841__index.html]

A genome-wide association study of atopic dermatitis identifies loci with overlapping effects on asthma and psoriasis — Supplementary Data 

# A genome-wide association study of atopic dermatitis identifies loci with overlapping effects on asthma and psoriasis

## Supplementary Data

Supplementary Data

**Files in this Data Supplement:**

- Supplementary Data - Docx file
