# Supplementary material for: A genome-wide association study of atopic dermatitis identifies loci with overlapping effects on asthma and psoriasis
Source: Hum Mol Genet. 2013 Jul 25;22(23):4841–56. doi: 10.1093/hmg/ddt317 (PMC3820131; doi:10.1093/hmg/ddt317)
Supplement: Supplementary Data [file supp_ddt317_ddt317supp.docx]

Human Molecular Genetics

Supplementary Material

**Title:** A genome-wide association study of Atopic Dermatitis identifies loci with overlapping effects on asthma and psoriasis

**Authors:** Stephan Weidinger∞^1^, Saffron A. G. Willis-Owen∞^2^, Yoichiro Kamatani∞^3^, Hansjörg Baurecht^1^, Nilesh Morar^2,4^, Liming Liang^5^, Pauline Edser^2^, Teresa Street^6^, Elke Rodriguez^1^, Grainne M O’Regan^7,8^, Paula Beattie^9^, Regina Fölster-Holst^10^, Andre Franke^11^, Natalija Novak^12^, Caoimhe M. Fahy^13^, Mårten C. G. Winge^14,15^, Michael Kabesch^16^, Thomas Illig^17,18^, Simon Heath^19^, Cilla Söderhäll^20^, Erik Melén^21, 22^, Göran Pershagen^21^, Juha Kere^23^, Maria Bradley^14,15^, Agne Lieden^15^, Magnus Nordenskjold^15^, John I. Harper^24,25^, W.H. Irwin McLean^26^, Sara J. Brown^26^, William O.C. Cookson^2^, G. Mark Lathrop^3,19,27^, Alan D. Irvine^7,8,13^ & Miriam F. Moffatt^*2^

∞ These authors contributed equally to this work

* *Corresponding Author*

*Address: National Heart and Lung Institute, Imperial College London SW3 6LY, UK.*

*Telephone: +44 (0) 20 7594 2942*

*Fax: +44 (0) 20 7351 8126*

*Email: m.moffatt@imperial.ac.uk*

**Cohort descriptions:**

German cases and controls

For the discovery phase, 682 German AD patients with childhood-onset and moderate-severe disease (OSCORAD>15) were obtained from the outpatient dermatology clinic of the University Hospital rechts der Isar, München, Germany ([1](#_ENREF_1)). The control group consisted of 751 individuals with no history of AD recruited as part of the cross sectional International Study of Asthma and Allergies in Childhood (ISAAC) ([2](#_ENREF_2)). For the replication stage, 1241 independent cases were obtained through the outpatient dermatology clinics based at the University Hospitals Munich, Bonn and Kiel, 49.8% of which had childhood AD. The dermatologist’s diagnosis of AD was made according to standard criteria in the presence of a chronic or chronically relapsing pruritic dermatitis with the typical morphology and distribution ([1](#_ENREF_1)). Subjects were classified as having asthma or allergic rhinitis when they reported a physician’s diagnosis of asthma or hayfever in the past. The controls group comprised 1178 German subjects with no history of AD from the KORA S4/F4 population-based survey ([3](#_ENREF_3)) and the ISAAC study.

Irish cases and controls

For the replication stage 631 AD cases were recruited from secondary/tertiary clinics in Our Lady’s Children’s Hospital Crumlin, Dublin and clinics in Glasgow, Dundee and Edinburgh (also in the NCRC collection). All patients all had early onset disease (<2 years; mean age at recruitment 2.8 years) with a mean AD severity score (Nottingham Eczema Severity Score; NESS) of 10.23 (SD 3.11).

The Irish controls comprised 996 subjects with no history of AD from a collection of 1237 blood donor volunteers of the Trinity Biobank.

Swedish cases and controls

422 unrelated (59.7% females; median 30.1 years, range 4-77 years) patients with moderate-to-severe AD were selected from a Swedish AD family material previously described ([4](#_ENREF_4), [5](#_ENREF_5)) All patients were investigated by a dermatologist performing clinical examination and recording medical history using a standardized questionnaire. The diagnosis of AD was made according to the UK Working Party’s diagnostic criteria. Severe AD (n= 135; 32 %) was defined as age of onset at/before 2 years of age, history of hospitalization for treatment and more than 3 affected sites upon recruitment. Moderate AD (n=287; 68 %) was defined as onset at/before 2 years of age and 1-3 affected sites upon examination and/or previous hospitalization for treatment. 999 controls with no history of AD were selected from the Swedish population-based birth cohort BAMSE ([6](#_ENREF_6)).

**References**

1 Naumann, A., Soderhall, C., Folster-Holst, R., Baurecht, H., Harde, V., Muller-Wehling, K., Rodriguez, E., Ruether, A., Franke, A., Wagenpfeil, S. *et al.* (2011) A comprehensive analysis of the COL29A1 gene does not support a role in eczema. *J. Allergy Clin. Immunol.*, **127**, 1187-1194 e1187.

2 Weiland, S. K., Bjorksten, B., Brunekreef, B., Cookson, W. O., von Mutius, E. and Strachan, D. P. (2004) Phase II of the International Study of Asthma and Allergies in Childhood (ISAAC II): rationale and methods. *Eur. Respir. J.*, **24**, 406-412.

3 Illig, T., Gieger, C., Zhai, G., Romisch-Margl, W., Wang-Sattler, R., Prehn, C., Altmaier, E., Kastenmuller, G., Kato, B. S., Mewes, H. W. *et al.* (2010) A genome-wide perspective of genetic variation in human metabolism. *Nat. Genet.*, **42**, 137-141.

4 Bradley, M., Kockum, I., Soderhall, C., Van Hage-Hamsten, M., Luthman, H., Nordenskjold, M. and Wahlgren, C. F. (2000) Characterization by phenotype of families with atopic dermatitis. *Acta Derm. Venereol.*, **80**, 106-110.

5 Bradley, M., Soderhall, C., Luthman, H., Wahlgren, C. F., Kockum, I. and Nordenskjold, M. (2002) Susceptibility loci for atopic dermatitis on chromosomes 3, 13, 15, 17 and 18 in a Swedish population. *Hum. Mol. Genet.*, **11**, 1539-1548.

6 Wickman, M., Kull, I., Pershagen, G. and Nordvall, S. L. (2002) The BAMSE project: presentation of a prospective longitudinal birth cohort study. *Pediatr. Allergy Immunol.*, **13 Suppl 15**, 11-13.

Tables

Table S1: Sample composition by phenotype
Abbreviations: DE (Germany), UK (United Kingdom), IE (Ireland), AD (Atopic Dermatitis).

Table S2: Filaggrin genotype availability and distribution across the discovery cohorts

Abbreviations: GWAS (Genome-Wide Association Study), *FLG* (Filaggrin), DE (Germany), UK (United Kingdom), IE (Ireland), AD (Atopic Dermatitis).

Table S3: Complete listing of the 47 discovery-phase SNPs prioritised for replication, including the three phenotypic models tested
Abbreviations: bp (Basepair), Chr (Chromosome), SNP (Single Nucleotide Polymorphism), StdErr (Standard Error), AD (Atopic Dermatitis).

Table S4: Replication phase results
Notes. Table S4 includes the results from the discovery phase AD screen, replication phase, and all phases combined. Markers that failed in the replication phase include a brief description outlining the type of failure. Abbreviations: HWE (Hardy Weinberg Equilibrium),DE (Germany), IE (Ireland), SWE (Sweden), CR (Call Rate).

Table S1

|  | ***AD*** | | ***AD plus asthma*** | | ***AD not asthma*** | |
| --- | --- | --- | --- | --- | --- | --- |
|  | ***UK + IE*** | ***DE*** | ***UK + IE*** | ***DE*** | ***UK + IE*** | ***DE*** |
| Cases (*n*) | 881 | 682 | 325 | 243 | 555 | 439 |
| Controls (*n*) | 3,442 | 612 | 3,442 | 612 | 3,442 | 612 |

Table S2

|  | | **GWAS** | **FLG** | | | | | | | |
| --- | --- | --- | --- | --- | --- | --- | --- | --- | --- | --- |
|  |  |  | ***FLG-2282DEL4*** | | | | ***FLG_R501X*** | | | |
|  |  |  | ***total*** | ***----/----*** | ***ACTG/----*** | ***ACTG/ACTG*** | ***total*** | ***A/A*** | ***G/A*** | ***G/G*** |
| UK+IE | Cases (*n*) | 881 | 829 | 15 | 146 | 668 | 854 | 6 | 162 | 686 |
|  | Controls (*n*) | 3442 | 515 | 0 | 22 | 493 | 516 | 0 | 24 | 492 |
| DE | Cases (*n*) | 682 | 629 | 6 | 97 | 526 | 646 | 0 | 64 | 582 |
|  | Controls (*n*) | 612 | 343 | 0 | 14 | 329 | 350 | 0 | 3 | 347 |

Table S3

|  |  |  | **AD** | | | | | **AD and asthma** | | | | | **AD not asthma** | | | | |
| --- | --- | --- | --- | --- | --- | --- | --- | --- | --- | --- | --- | --- | --- | --- | --- | --- | --- |
| SNP | Chr | Position (Bp) | Allele1 | Allele2 | Effect | StdErr | P-value | Allele1 | Allele2 | Effect | StdErr | P-value | Allele1 | Allele2 | Effect | StdErr | P-value |
| rs17006471 | 2 | 70922131 | t | c | -0.25 | 0.05 | 1.00E-06 | t | c | -0.28 | 0.07 | 1.05E-04 | t | c | -0.24 | 0.06 | 1.62E-04 |
| rs1469621 | 2 | 137592425 | a | g | 0.32 | 0.06 | 3.88E-09 | a | g | 0.35 | 0.08 | 4.33E-06 | a | g | 0.31 | 0.07 | 4.24E-06 |
| rs7581601 | 2 | 203497786 | a | c | 0.26 | 0.06 | 4.98E-06 | a | c | 0.31 | 0.08 | 1.42E-04 | a | c | 0.20 | 0.07 | 3.89E-03 |
| rs162907 | 5 | 131608051 | a | g | -0.26 | 0.05 | 1.37E-06 | a | g | -0.25 | 0.07 | 7.00E-04 | a | g | -0.26 | 0.07 | 9.15E-05 |
| rs2897443 | 5 | 131957493 | t | g | 0.30 | 0.06 | 1.27E-06 | t | g | 0.32 | 0.08 | 1.78E-04 | t | g | 0.29 | 0.08 | 1.41E-04 |
| rs6871536 | 5 | 131997773 | t | c | -0.30 | 0.06 | 7.27E-07 | t | c | -0.33 | 0.08 | 7.97E-05 | t | c | -0.28 | 0.07 | 1.67E-04 |
| rs2158177 | 5 | 132011957 | a | g | -0.49 | 0.08 | 2.65E-10 | a | g | -0.58 | 0.10 | 3.66E-08 | a | g | -0.47 | 0.10 | 1.11E-06 |
| rs1295686 | 5 | 132023742 | t | c | 0.35 | 0.06 | 2.66E-08 | t | c | 0.40 | 0.09 | 2.38E-06 | t | c | 0.30 | 0.08 | 1.00E-04 |
| rs20541 | 5 | 132023863 | a | g | 0.35 | 0.06 | 1.79E-08 | a | g | 0.41 | 0.09 | 1.61E-06 | a | g | 0.30 | 0.08 | 8.59E-05 |
| rs2243204 | 5 | 132027393 | t | c | 0.44 | 0.09 | 1.03E-06 | t | c | 0.58 | 0.12 | 6.47E-07 | t | c | 0.32 | 0.11 | 4.11E-03 |
| rs17076328 | 5 | 173076114 | a | g | 0.31 | 0.06 | 8.38E-07 | a | g | 0.30 | 0.09 | 7.64E-04 | a | g | 0.32 | 0.08 | 5.34E-05 |
| rs6909636 | 6 | 31454578 | a | g | 0.27 | 0.06 | 3.84E-06 | a | g | 0.28 | 0.08 | 6.09E-04 | a | g | 0.28 | 0.07 | 1.25E-04 |
| rs2251396 | 6 | 31472686 | a | g | 0.28 | 0.06 | 2.65E-06 | a | g | 0.40 | 0.08 | 5.91E-07 | a | g | 0.18 | 0.07 | 1.54E-02 |
| rs2248372 | 6 | 31554445 | a | g | -0.30 | 0.06 | 3.49E-07 | a | g | -0.34 | 0.08 | 4.05E-05 | a | g | -0.26 | 0.07 | 2.96E-04 |
| rs2905731 | 6 | 31565259 | a | g | 0.38 | 0.08 | 2.57E-06 | a | g | 0.22 | 0.11 | 5.01E-02 | a | g | 0.51 | 0.10 | 6.13E-07 |
| rs2844509 | 6 | 31618903 | a | g | 0.30 | 0.07 | 5.64E-06 | a | g | 0.21 | 0.09 | 2.28E-02 | a | g | 0.36 | 0.08 | 8.82E-06 |
| rs3093662 | 6 | 31652168 | a | g | 0.50 | 0.11 | 2.59E-06 | a | g | 0.42 | 0.15 | 4.57E-03 | a | g | 0.56 | 0.13 | 1.72E-05 |
| rs9267502 | 6 | 31661173 | a | g | -0.51 | 0.11 | 1.92E-06 | a | g | -0.43 | 0.15 | 5.11E-03 | a | g | -0.59 | 0.13 | 1.11E-05 |
| rs9378200 | 6 | 31680906 | t | c | 0.55 | 0.11 | 1.90E-06 | t | c | 0.42 | 0.16 | 7.25E-03 | t | c | 0.66 | 0.15 | 5.25E-06 |
| rs9368699 | 6 | 31910520 | t | c | 0.64 | 0.14 | 7.61E-06 | t | c | 0.56 | 0.21 | 6.24E-03 | t | c | 0.70 | 0.18 | 8.08E-05 |
| rs4151657 | 6 | 32025519 | t | c | -0.29 | 0.06 | 1.56E-06 | t | c | -0.25 | 0.08 | 2.32E-03 | t | c | -0.31 | 0.07 | 2.98E-05 |
| rs2280774 | 6 | 32036670 | a | g | 0.31 | 0.06 | 2.51E-07 | a | g | 0.28 | 0.08 | 6.44E-04 | a | g | 0.32 | 0.07 | 1.43E-05 |
| rs6941112 | 6 | 32054593 | a | g | 0.31 | 0.06 | 1.98E-07 | a | g | 0.28 | 0.08 | 6.28E-04 | a | g | 0.34 | 0.07 | 4.59E-06 |
| rs6474 | 6 | 32114865 | a | g | 0.37 | 0.06 | 1.61E-09 | a | g | 0.36 | 0.08 | 1.39E-05 | a | g | 0.37 | 0.07 | 7.93E-07 |
| rs17421133 | 6 | 32118251 | a | t | 0.35 | 0.06 | 3.76E-09 | a | t | 0.33 | 0.08 | 6.06E-05 | a | t | 0.37 | 0.07 | 5.57E-07 |
| rs12198173 | 6 | 32134786 | a | g | -0.43 | 0.10 | 5.56E-06 | a | g | -0.43 | 0.14 | 2.01E-03 | a | g | -0.48 | 0.12 | 4.64E-05 |
| rs2239689 | 6 | 32138262 | a | g | 0.35 | 0.06 | 7.74E-09 | a | g | 0.33 | 0.08 | 5.65E-05 | a | g | 0.36 | 0.07 | 6.76E-07 |
| rs12211410 | 6 | 32157401 | t | c | -0.43 | 0.10 | 6.35E-06 | t | c | -0.42 | 0.14 | 2.20E-03 | t | c | -0.48 | 0.12 | 4.64E-05 |
| rs13199524 | 6 | 32174743 | t | c | -0.45 | 0.10 | 3.81E-06 | t | c | -0.42 | 0.14 | 2.55E-03 | t | c | -0.50 | 0.12 | 2.61E-05 |
| rs12153855 | 6 | 32182782 | t | c | 0.43 | 0.09 | 9.46E-07 | t | c | 0.39 | 0.13 | 1.95E-03 | t | c | 0.49 | 0.11 | 7.99E-06 |
| rs8111 | 6 | 32191153 | t | c | 0.35 | 0.06 | 1.12E-08 | t | c | 0.31 | 0.08 | 2.40E-04 | t | c | 0.39 | 0.07 | 1.44E-07 |
| rs204999 | 6 | 32217957 | a | g | 0.30 | 0.07 | 5.82E-06 | a | g | 0.25 | 0.09 | 6.54E-03 | a | g | 0.37 | 0.08 | 8.23E-06 |
| rs2071286 | 6 | 32287874 | t | c | 0.30 | 0.07 | 8.17E-06 | t | c | 0.22 | 0.09 | 1.31E-02 | t | c | 0.39 | 0.08 | 2.05E-06 |
| rs3134929 | 6 | 32300085 | c | g | 0.43 | 0.07 | 2.64E-09 | c | g | 0.50 | 0.10 | 1.70E-06 | c | g | 0.38 | 0.09 | 1.32E-05 |
| rs3104395 | 6 | 32793404 | a | g | -0.40 | 0.09 | 3.21E-06 | a | g | -0.39 | 0.12 | 1.36E-03 | a | g | -0.41 | 0.11 | 1.18E-04 |
| rs4750646 | 10 | 15494474 | a | g | 0.28 | 0.06 | 2.72E-06 | a | g | 0.21 | 0.08 | 8.70E-03 | a | g | 0.35 | 0.07 | 2.18E-06 |
| rs10994443 | 10 | 62395518 | a | g | -0.48 | 0.10 | 5.10E-07 | a | g | -0.32 | 0.13 | 1.73E-02 | a | g | -0.56 | 0.12 | 3.11E-06 |
| rs2393902 | 10 | 64039007 | t | c | -0.26 | 0.06 | 2.31E-06 | t | c | -0.29 | 0.08 | 1.25E-04 | t | c | -0.26 | 0.07 | 1.46E-04 |
| rs10995245 | 10 | 64061381 | a | g | 0.24 | 0.05 | 7.18E-06 | a | g | 0.31 | 0.07 | 2.13E-05 | a | g | 0.21 | 0.07 | 1.80E-03 |
| rs10761652 | 10 | 64067662 | a | g | 0.27 | 0.06 | 6.54E-06 | a | g | 0.25 | 0.08 | 3.34E-03 | a | g | 0.31 | 0.07 | 3.33E-05 |
| rs593982 | 11 | 65269683 | t | c | -0.40 | 0.09 | 7.71E-06 | t | c | -0.33 | 0.13 | 8.01E-03 | t | c | -0.42 | 0.11 | 1.96E-04 |
| rs7130588 | 11 | 75948331 | a | g | -0.26 | 0.05 | 5.81E-07 | a | g | -0.27 | 0.07 | 1.71E-04 | a | g | -0.26 | 0.06 | 3.94E-05 |
| rs2155219 | 11 | 75976842 | t | g | 0.31 | 0.05 | 8.17E-09 | t | g | 0.35 | 0.07 | 3.67E-06 | t | g | 0.30 | 0.07 | 6.01E-06 |
| rs17101056 | 12 | 63806743 | c | g | -0.39 | 0.09 | 7.34E-06 | c | g | -0.51 | 0.11 | 7.76E-06 | c | g | -0.22 | 0.11 | 4.17E-02 |
| rs11175919 | 12 | 64466544 | a | g | -0.78 | 0.17 | 4.09E-06 | a | g | -0.93 | 0.26 | 2.88E-04 | a | g | -0.62 | 0.20 | 2.46E-03 |
| rs11160835 | 14 | 104658689 | t | c | 0.30 | 0.06 | 3.67E-07 | t | c | 0.26 | 0.08 | 1.44E-03 | t | c | 0.33 | 0.07 | 7.58E-06 |
| rs4782695 | 16 | 83827911 | a | g | -0.48 | 0.11 | 4.70E-06 | a | g | -0.37 | 0.14 | 1.07E-02 | a | g | -0.59 | 0.13 | 4.97E-06 |

Table S4

|  |  |  |  |  | ***Screen*** | | ***Replication*** | | ***Combined*** | |
| --- | --- | --- | --- | --- | --- | --- | --- | --- | --- | --- |
| ***SNP*** | ***Chr*** | ***Position (Bp)*** | ***A1*** | ***A2*** | ***OR(95%CI)*** | ***P*** | ***OR(95%CI)*** | ***P*** | ***OR(95%CI)*** | ***P*** |
| rs17006471 | 2 | 70922131 | T | C | 0.778 (0.704-0.86) | 1.00E-06 | 0.955(0.727-1.254) | 7.43E-01 | 0.802 (0.743-0.865) | 1.11E-08 |
| rs1469621 | 2 | 137592425 | A | G | 1.383 (1.241-1.54) | 3.88E-09 | 1.051(0.915-1.208) | 4.79E-01 | 1.044 (0.837-1.302) | 7.04E-01 |
| rs7581601 | 2 | 203497786 | A | C | 1.298 (1.16-1.451) | 4.98E-06 | 1.051(0.953-1.16) | 3.18E-01 | 1.19 (0.999-1.416) | 5.10E-02 |
| rs162907 | 5 | 131608051 | A | G | 0.77 (0.693-0.856) | 1.37E-06 | 0.899(0.828-0.976) | 1.00E-02 | 0.861 (0.782-0.947) | 2.21E-03 |
| rs2897443 | 5 | 131957493 | T | G | 1.35 (1.196-1.525) | 1.27E-06 | 1.284(1.171-1.408) | 1.20E-07 | 1.308 (1.215-1.407) | 8.95E-13 |
| rs6871536 | 5 | 131997773 | T | C | 0.738 (0.655-0.832) | 7.27E-07 | 0.747(0.678-0.822) | 3.42E-09 | 0.743 (0.69-0.799) | 2.11E-15 |
| rs2158177 | 5 | 132011957 | A | G | 0.611 (0.524-0.712) | 2.65E-10 | 0.738(0.672-0.811) | 2.49E-10 | 0.704 (0.634-0.782) | 5.90E-11 |
| rs1295686 | 5 | 132023742 | T | C | 1.42 (1.255-1.606) | 2.66E-08 | 1.362(1.24-1.496) | 9.80E-11 | 1.383 (1.283-1.49) | 1.65E-17 |
| rs20541 | 5 | 132023863 | A | G | 1.426 (1.26-1.613) | 1.79E-08 | 1.365(1.202-1.55) | 1.56E-06 | 1.39 (1.278-1.512) | 1.93E-14 |
| rs2243204 | 5 | 132027393 | T | C | 1.548 (1.299-1.844) | 1.03E-06 | 1.123(0.769-1.639) | 5.47E-01 | 1.431 (1.218-1.682) | 1.30E-05 |
| rs17076328 | 5 | 173076114 | A | G | 1.368 (1.208-1.55) | 8.38E-07 | 0.973(0.864-1.097) | 6.59E-01 | 1.068 (0.873-1.307) | 5.21E-01 |
| rs6909636 | 6 | 31454578 | A | G | 1.312 (1.169-1.473) | 3.84E-06 | 1.119(1.034-1.21) | 5.70E-03 | 1.178 (1.066-1.301) | 1.33E-03 |
| rs2251396 | 6 | 31472686 | A | G | 1.325 (1.178-1.49) | 2.65E-06 | 1.23(1.109-1.365) | 1.09E-04 | 1.267 (1.174-1.368) | 1.28E-09 |
| rs2248372 | 6 | 31554445 | A | G | 0.743 (0.663-0.833) | 3.49E-07 | 0.819(0.719-0.932) | 2.50E-03 | 0.801 (0.69-0.929) | 3.47E-03 |
| rs2905731 | 6 | 31565259 | A | G | 1.467 (1.251-1.721) | 2.57E-06 | DE:HWE,CR; IE:failed | | - | - |
| rs2844509 | 6 | 31618903 | A | G | 1.345 (1.184-1.529) | 5.64E-06 | 1.624(1.249-2.112) | 3.00E-04 | 1.312 (1.215-1.417) | 4.33E-12 |
| rs3093662 | 6 | 31652168 | A | G | 1.645 (1.337-2.025) | 2.59E-06 | 1.283(1.069-1.539) | 7.50E-03 | 1.421 (1.218-1.658) | 8.07E-06 |
| rs9267502 | 6 | 31661173 | A | G | 0.599 (0.485-0.739) | 1.92E-06 | 0.786(0.67-0.921) | 2.80E-03 | 0.728 (0.605-0.877) | 8.22E-04 |
| rs9378200 | 6 | 31680906 | T | C | 1.725 (1.378-2.159) | 1.90E-06 | 1.338(1.119-1.599) | 1.40E-03 | 1.452 (1.224-1.723) | 1.84E-05 |
| rs9368699 | 6 | 31910520 | T | C | 1.894 (1.432-2.505) | 7.61E-06 | 1.531(1.201-1.952) | 5.81E-04 | 1.677 (1.396-2.014) | 3.19E-08 |
| rs4151657 | 6 | 32025519 | T | C | 0.75 (0.666-0.843) | 1.56E-06 | 0.934(0.854-1.022) | 1.38E-01 | 0.869 (0.758-0.997) | 4.57E-02 |
| rs2280774 | 6 | 32036670 | A | G | 1.363 (1.211-1.533) | 2.51E-07 | 1.144(0.996-1.313) | 5.69E-02 | 1.222 (1.059-1.411) | 6.08E-03 |
| rs6941112 | 6 | 32054593 | A | G | 1.366 (1.215-1.537) | 1.98E-07 | 1.178(1.081-1.284) | 2.00E-04 | 1.234 (1.12-1.359) | 1.95E-05 |
| rs6474 | 6 | 32114865 | A | G | 1.444 (1.282-1.627) | 1.61E-09 | DE:HWE,CR; IE:HWE; SWE:HWE | | - | - |
| rs17421133 | 6 | 32118251 | A | T | 1.426 (1.267-1.604) | 3.76E-09 | DE:HWE,CR; SWE:HWE,CR | | - | - |
| rs12198173 | 6 | 32134786 | A | G | 0.649 (0.539-0.782) | 5.56E-06 | 0.676(0.571-0.801) | 5.32E-06 | 0.664 (0.586-0.752) | 1.33E-10 |
| rs2239689 | 6 | 32138262 | A | G | 1.414 (1.257-1.59) | 7.74E-09 | 1.124(0.986-1.282) | 8.10E-02 | 1.21 (1.024-1.429) | 2.49E-02 |
| rs12211410 | 6 | 32157401 | T | C | 0.651 (0.54-0.784) | 6.35E-06 | 0.719(0.619-0.834) | 1.56E-05 | 0.692 (0.615-0.777) | 5.78E-10 |
| rs13199524 | 6 | 32174743 | T | C | 0.639 (0.528-0.773) | 3.81E-06 | 0.63(0.514-0.772) | 8.96E-06 | 0.641 (0.564-0.728) | 8.01E-12 |
| rs12153855 | 6 | 32182782 | T | C | 1.541 (1.296-1.832) | 9.46E-07 | 1.665(1.353-2.05) | 1.46E-06 | 1.581 (1.405-1.779) | 2.96E-14 |
| rs8111 | 6 | 32191153 | T | C | 1.416 (1.257-1.596) | 1.12E-08 | 1.176(0.98-1.411) | 8.17E-02 | 1.236 (1.047-1.46) | 1.22E-02 |
| rs204999 | 6 | 32217957 | A | G | 1.352 (1.187-1.541) | 5.82E-06 | 1.221(1.052-1.418) | 8.30E-03 | 1.251 (1.072-1.46) | 4.41E-03 |
| rs2071286 | 6 | 32287874 | T | C | 1.345 (1.181-1.532) | 8.17E-06 | 1.038(0.914-1.179) | 5.74E-01 | 1.136 (0.948-1.361) | 1.69E-01 |
| rs3134929 | 6 | 32300085 | C | G | 1.532 (1.331-1.763) | 2.64E-09 | 1.241(1.121-1.374) | 2.88E-05 | 1.33 (1.174-1.505) | 7.06E-06 |
| rs3104395 | 6 | 32793404 | A | G | 0.667 (0.563-0.791) | 3.21E-06 | DE:monomorph; IE:failed; SWE:monomorph | | - | - |
| rs4750646 | 10 | 15494474 | A | G | 1.322 (1.176-1.485) | 2.72E-06 | 0.955(0.869-1.049) | 3.35E-01 | 1.111 (0.792-1.559) | 5.41E-01 |
| rs10994443 | 10 | 62395518 | A | G | 0.618 (0.512-0.745) | 5.10E-07 | 0.967(0.859-1.087) | 5.71E-01 | 0.868 (0.686-1.097) | 2.36E-01 |
| rs2393902 | 10 | 64039007 | T | C | 0.768 (0.688-0.857) | 2.31E-06 | 0.863(0.768-0.971) | 1.39E-02 | 0.833 (0.756-0.917) | 2.00E-04 |
| rs10995245 | 10 | 64061381 | A | G | 1.269 (1.144-1.409) | 7.18E-06 | 1.183(0.988-1.417) | 6.70E-02 | 1.204 (1.058-1.37) | 4.92E-03 |
| rs10761652 | 10 | 64067662 | A | G | 1.309 (1.164-1.471) | 6.54E-06 | 1.023(0.866-1.209) | 7.83E-01 | 1.092 (0.926-1.288) | 2.95E-01 |
| rs593982 | 11 | 65269683 | T | C | 0.667 (0.559-0.797) | 7.71E-06 | 0.874(0.725-1.053) | 1.54E-01 | 0.812 (0.675-0.978) | 2.82E-02 |
| rs7130588 | 11 | 75948331 | A | G | 0.77 (0.694-0.853) | 5.81E-07 | 0.794(0.708-0.889) | 7.44E-05 | 0.778 (0.727-0.833) | 4.49E-13 |
| rs2155219 | 11 | 75976842 | T | G | 1.36 (1.225-1.51) | 8.17E-09 | 1.319(1.205-1.444) | 1.66E-09 | 1.323 (1.224-1.429) | 1.61E-12 |
| rs17101056 | 12 | 63806743 | C | G | 0.677 (0.57-0.803) | 7.34E-06 | 0.974(0.846-1.122) | 7.17E-01 | 0.88 (0.711-1.089) | 2.40E-01 |
| rs11175919 | 12 | 64466544 | A | G | 0.458 (0.328-0.638) | 4.09E-06 | 1.305(0.896-1.901) | 1.67E-01 | 0.996 (0.534-1.859) | 9.91E-01 |
| rs11160835 | 14 | 104658689 | T | C | 1.352 (1.204-1.518) | 3.67E-07 | 0.976(0.88-1.083) | 6.47E-01 | 1.172 (0.882-1.556) | 2.73E-01 |
| rs4782695 | 16 | 83827911 | A | G | 0.618 (0.503-0.759) | 4.70E-06 | 0.927(0.826-1.04) | 1.97E-01 | 0.834 (0.676-1.028) | 8.92E-02 |
